# Long non-coding RNA Homeobox D gene cluster antisense growth-associated long noncoding RNA/microRNA-182-5p/Homeobox protein A10 alleviates postmenopausal osteoporosis via accelerating osteoblast differentiation of bone marrow mesenchymal stem cells

**DOI:** 10.1186/s13018-023-04203-8

**Published:** 2023-09-26

**Authors:** YeJian Huang, MingGao Tao, ShiXian Yan, XueMing He

**Affiliations:** 1 Department of Spine and Traumatology, The Affiliated Lianyungang Oriental Hospital of Xuzhou Medical University, Lianyungang City, 221004 Jiangsu Province China; 2 Department of Center for Clinical Research and Translational Medicine, The Affiliated Lianyungang Oriental Hospital of Xuzhou Medical University, No. 379, Tongshan Road, Dongdianzi, Long District, Lianyungang City, 221004 Jiangsu Province China

**Keywords:** Postmenopausal osteoporosis, Bone marrow mesenchymal stem cells, HAGLR, MicroRNA-182-5p, Homeobox protein A10

## Abstract

**Background:**

Studies have illuminated that long non-coding RNA (lncRNA) influences bone cell differentiation and formation. Nevertheless, whether lncRNA Homeobox D gene cluster antisense growth-associated long noncoding RNA (HAGLR) was implicated in postmenopausal osteoporosis (PMOP) was yet uncertain.

**Purpose:**

The research was to explore HAGLR’s role in the osteogenic differentiation (OD) process of bone marrow mesenchymal stem cells (BMSCs).

**Methods:**

BMSCs were isolated from mouse bone marrow tissues and identified by electron microscope and flow cytometry. HAGLR, microRNA (miR)-182-5p, and homeobox protein A10 (Hoxa10) levels in BMSCs were detected. Mouse BMSC OD process was induced, and calcium deposition and alkaline phosphatase content were analyzed, as well as expressions of runt-related transcription factor 2, osteopontin, and osteocalcin, and cell apoptosis. Bilateral ovaries were resected from mice to construct the ovariectomized model and bone mineral density, maximum bending stress, maximum load, and elastic modulus of the femur were tested, and the femur was histopathologically evaluated. Chondrocyte apoptosis in the articular cartilage of mice was analyzed. Analysis of the interaction of HAGLR, miR-182-5p with Hoxa10 was conducted.

**Results:**

HAGLR and Hoxa10 were down-regulated and miR-182-5p was elevated in PMOP patients. During the BMSC OD process, HAGLR and Hoxa10 levels were suppressed, while miR-182-5p was elevated. Promotion of HAGLR or suppression of miR-182-5p accelerated OD of BMSCs. Inhibition of miR-182-5p reversed the inhibitory effect of HAGLR on BMSC OD. In in vivo experiments, up-regulating HAGLR alleviated PMOP, while silencing Hoxa10 reversed the effects of upregulating HAGLR. HAGLR performed as a sponge for miR-182-5p, while miR-182-5p targeted Hoxa10.

**Conclusion:**

In general, HAGLR boosted the OD process of BMSCs and relieved PMOP via the miR-182-5p/Hoxa10 axis. These data preliminarily reveal the key role of HAGLR in PMOP, and the research results have a certain reference for the treatment of PMOP.

## Introduction

Postmenopausal Osteoporosis (PMOP) is a popular systemic chronic metabolic disease that poses a serious threat to women’s health around the world [1]. PMOP is more common in women 5–10 years after menopause and is due to decreased PM estrogen that accelerates osteoclast formation and bone resorption [2–4]. While the imbalance of bone resorption and formation leads to decreased bone mineral density (BMD) and bone microstructure destruction [5]. Early PMOP is hard to be detected and no distinct symptoms are presented before the fracture [6]. The treatment and prevention of osteoporotic fractures, which result in increased disability, mortality, and health care costs, have important clinical and public health implications [7–10]. Presently, detailed molecular mechanisms and treatment strategies of PMOP remain uncertain.

As reported, the dysfunction of bone marrow mesenchymal stem cells (BMSCs) is the crux of OP [11]. The differentiation of mesenchymal stem cells into osteoblasts and the differentiation of circulating monocytes into osteoclasts exert a crucial role in bone metabolic balance [12]. Studies have elaborated that BMSCs are linked with decreased osteogenesis and elevated oxidative stress [13]. BMSCs become a hot topic in bone regeneration research because of their excellent osteogenic potential and abundant sources, but their clinical application is restrained because of elevated cost and decreased efficiency [14]. It is of momentous value to explore the molecular mechanism of BMSCs differentiation for PMOP cure.

Long non-coding RNA (lncRNA), a conserved specific non-coding RNA, participates in multiple biological processes covering signal transduction and cell growth, etc. [15]. As reported, LncRNAs are implicated in BMSCs differentiation [16]. For instance, lncRNA MEG3 restrains the osteogenic differentiation (OD) of BMSCs in PMOP via targeting microRNA (miR)-133a-3p [17]. LncRNA Homeobox D gene cluster antisense growth-associated long noncoding RNA (HAGLR) has been testified to exert a crucial role in femoral neck fracture healing [18]. Nevertheless, its action in PMOP is uncertain.

Several mechanisms for lncRNA regulation have been characterized, including histone modification [19], transcription factor regulation [20], alternative splicing [21], and competing endogenous RNA (ceRNA) of miRNAs [22, 23]. By sponging miRNAs, lncRNAs protect corresponding mRNAs from being silenced. miRNAs, a set of short non-coding RNAs, modulate biological processes such as cell differentiation [24]. miRNAs regulate gene expression at the post-transcriptional level by inhibiting the translation of messenger RNA (mRNA) or accelerating the degradation of mRNA, thereby regulating many physiological and pathological processes [25]. miRNAs are implicated in PMOP and are regarded as latent curative targets [26]. For instance, miR-218-5p alleviates PMOP via accelerating osteoblast differentiation of BMSCs [27]. Bioinformation website analysis found that miR-182-5p was the downstream target of HAGLR. MiR-182-5p, a member of the miR-183/96/182 cluster, has been reported as a tumor oncogene or suppressor gene in diversified cancers [28]. Nevertheless, no research has illuminated its action in PMOP.

The study was to explore the action and molecular mechanism of HAGLR in PMOP. The research results uncovered that HAGLR regulates the differentiation of BMSCs via the miR-182-5p/Homeobox protein A10 (Hoxa10) axis, thereby influencing PMOP progression.

## Materials and methods

### Clinical samples

PMOP patients (*n* = 55) and healthy PM women (*n* = 55) in The Affiliated Lianyungang Oriental Hospital of Xuzhou Medical University were enrolled, and peripheral blood samples were collected. Lumbar spine bone mineral density (L1-L4) was assessed according to standard operating instructions using a dual-energy X-ray bone densimeter (DXA) (Hologic Discovery Wi, Hologic, USA). T-score is the standard deviation of bone mineral density relative to the mean. Osteoporosis is defined as bone mineral density of the lumbar spine (L1-L4) with a T-score ≤ -2.5. The inclusion criteria are as follows: (1) age ≥ 50 years; (2) Menopause ≥ 1 year; (3) Sign informed consent before participating in the study. Exclusion criteria are as follows: (1) any comorbidities that may significantly affect bone metabolisms, such as thyroid disease, diabetes, cancer, kidney disease, or ankylosing spondylitis; (2) previous anti-osteoporosis medication or hormone therapy (vitamin D and/or calcium supplements are allowed), such as estrogen or glucocorticoids; (3) A history of smoking or alcohol dependence within the past year. The research was approved by the Ethics Committee of The Affiliated Lianyungang Oriental Hospital of Xuzhou Medical University.

### Isolation, culture, and induction of BMSCs

Bone marrow was obtained from mice (Guangdong Medical Laboratory Animal Center, Guangdong, China). BMSCs were isolated by the whole bone marrow adhesion method and cultured in a-MEM medium (HyClone) containing 10% fetal bovine serum (FBS) (Gibco) and 1% penicillin (HyClone). After passages, BMSCs were identified by flow cytometry to detect surface markers CD34, CD45, CD73, and CD90.

BMSCs were cultured in 6-well plates and added an osteogenic induction medium containing Dulbecco’s Modified Eagle Medium, 10% FBS, 0.1 mg/mL dexamethasone, 50 mg/mL ascorbic acid, and 10 mmol/L glycerophosphate. The medium was replaced every 3 days.

### Cell culture and transfection

MiR-182-5p, anti-miR-182-5p, sh-HAGLR/Hoxa10, and their respective negative controls (NCs) were provided by GenePharma (Shanghai, China). Transfection of BMSCs was done with Lipofectamine 3000 reagent (L3000015, Invitrogen) in the light of the manufacturer’s instructions. AD-HAGLR-EGFP and NC adenovirus (AD-EGFP) were produced by Han Biotechnology (Shanghai, China).

### Alizarin red S staining

When the confluence of BMSCs reached about 100%, osteogenic differentiation was induced by adding osteogenic induction medium. After 14 days,cells were fixed with 95% cold ethanol for 25 min and air-dried. Alizarin Red S (40 mmol/L; A5533, Sigma Aldrich, USA) was dissolved in dH_2_O, and cells were stained with the prepared solution at 25℃ for 30 min. Then, 10% (w/v) cetylpyridine chloride (HC0502, HEROCHEM, Shanghai, China) was prepared and used for decolorization. The absorbance at 560 nm was read [29].

### Alkaline phosphatase (ALP) staining

Osteoblasts were detached with trypsin and seeded in a 24-well plate. Then, cells were treated with propanol (15 min), incubation solution (6 h), cobalt nitrate (15 min), and ammonium sulfide (5 min) (all 200 μL). Optical density at 490 nm was read on a microplate reader (Varioskan LUX; Thermo Fisher Scientific) [30].

### Flow cytometry

To assess cell apoptosis, 2 mL cell suspension at 1 × 10^5^ cells/mL was seeded into a 6-well plate. After 72 h, cells were centrifuged at 1000 rpm for 3 min and tested by an Apoptosis Detection Kit (559763, BD Biosciences, USA). Cell staining was done using Fluorescein isothiocyanate-Annexin V and propidium iodide, and analysis of cell apoptosis was performed using flow cytometry (BD Biosciences). Cell QuestPro software (BD Biosciences) was used for apoptosis analysis [31].

### Construction of the PMOP mouse model

Fifty Balb/c mice were randomly divided into the ovariectomized model (OVX) and the sham operation control (sham) groups. The OVX mice were anesthetized with 5% ketamine and sterilized normally. The ovaries on both sides of the mice were removed in a biologically clean environment. After complete hemostasis, the abdominal wound was sutured. Only a small amount of fat was removed in sham surgery. Two groups of mice were fed separately with free food and water. All experiments on mice met the standard guidelines for the use of animals in scientific research.

### PMOP mouse grouping

Four weeks after ovariectomy, PMOP mice were injected with AD-HAGLR-EGFP, NC adenovirus (AD-EGFP), and sh-Hoxa10. On days 1 to 3 of weeks 1 and 4, mice (*n* = 8) were administered at a dose of 7 mg/kg via the caudal vein. PMOP mice injected with normal saline were regarded as controls (PMOP; *n* = 8). Six weeks after the first injection, the bilateral femurs were collected from mice.

### Analysis of BMD and biomechanical parameters

BMD levels in the left femur of mice were measured using a Lunar DPX-IQ dual-energy X-ray absorptiometry with a PIXImus II absorptiometry (Lunar Corporation, Madison, WI). Elastic modulus, maximum load, and maximum bending stress were tested according to the requirements of the three-point bending test using a computer-controlled mechanical testing machine (SANS-10404043, Shenzhen, China). The sample distance is 23 mm and the plunger speed is 2.0 mm/min [32].

### Hematoxylin and Eosin (HE) staining

Tibias of mice were fixed with paraformaldehyde (P0099, Beyotime Biotechnology, China) for 1 week and rinsed 3 times to remove excess paraformaldehyde. Tibias were embedded in paraffin and cut into 5 μm sections, followed by treatments with xylene and graded ethanol. HE staining was performed using Hematoxylin and Eosin (G1120, Solarbio, China) according to the manufacturer's instructions. The morphology of the tibias was observed under a microscope. HE staining was performed on the left hind leg, and the mice in each group were examined on the same side [33].

### TdT-mediated dUTP-biotin nick end-labeling (TUNEL) staining

To test cell apoptosis, the tissues were treated with 2-methoxyethyl acetate and then incubated in 10 mM citrate buffer (pH 6.3) at 90℃ for 15 min. Subsequently, the sections were incubated with 0.5% pepsin at 37℃ for 30 min and detected by an in situ cell death detection kit (12,156,792,910, Roche, USA). Sections were stained with diaminobenzidine (Sigma) after incubation with POD at 37℃ for 1 h and then examined under a light microscope. TUNEL-positive cells were counted in 5 random fields [34].

### Reverse transcription-quantitative polymerase chain reaction (RT-qPCR)

Total RNA was extracted from tissues or cells using TRIzol (15,596,026, Invitrogen) and reverse-transcribed using the PrimeScript RT Master Mix kit (RR036B, Takara, Dalian, China) according to the manufacturer's recommended instructions. Then, RT-qPCR was performed in the ABI Prism 7900HT sequence detection system (Applied Biosystems) using SYBR Green Real Time PCR Master Mix (QPK-201, Toyobo, Osaka, Japan). Gene quantification was performed using the 2^−ΔΔCT^ method. Glyceraldehyde-3-phosphate dehydrogenase (GAPDH) and U6 were used as loading controls, respectively. The primer sequences for RT-qPCR were presented in Table 1 [35]. The agarose gel images are presented in the supplementary Fig. 1.

**Table 1:** RT-qPCR primer sequence

Genes		Forward (5′-3′)	Reverse (5′-3′)
HAGLR	Human	AGAAGTCTCGGGAACCTCCA	ACAGTGTGTTACCGCAGGAG
	Mouse	CCACGCTAGGAGTGAGTGTG	AAGTGTCAGGTTGGGGGTTC
Hoxa10	Human	AGAGATTAGCCGCAGCGTCC	TTCCTGGGCAGAGCCTGAAG
	Mouse	AGAGATTAGCCGCAGCGTCC	TTCCTGGGCAGAGCCTGAAG
OPN	Human	GATGGCCGAGGTGATAGTGT	GTGGGTTTCAGCACTCTGGT
OCN	Human	GGCAGCGAGGTAGTGAAGAG	CTAGACCGGGCCGTAGAAG
Runx2	Human	GAATGCACTACCCAGCCAC	TGGCAGGTACGTGTGGTAG
GAPDH	Human	CACCCACTCCTCCACCTTTG	CCACCACCCTGTTGCTGTAG
	Mouse	CATCAACGGGAAGCCCATC	CTCGTGGTTCACACCCATC
miR-182-5p	Human	CGGACTTTGGCAATGGTAGAACT	GCAGGGTCCGAGGTATTC
U6	Human	CTCGCTTCGGCAGCACA	AACGCTTCACGAATTTGCGT

### Western blot analysis

Radioimmunoprecipitation assay lysis buffer (P0013B, Beyotime) was added to extract the total protein. Subsequently, proteins were separated by electrophoresis and electroblotted onto polyvinylidene fluoride membranes (Millipore). After incubation with 5% skim milk for 2 h, the membrane was reacted overnight with primary antibodies Hoxa10 (ab191470), Runt-related transcription factor 2 (RUNX2) (ab76956), osteopontin (OPN) (ab214050) (1: 1000), Osteocalcin (OCN) (ab93876, 1: 500) and GAPDH (ab8245, 1: 2000, Abcam). Membranes were incubated with the corresponding secondary antibody for 2 h and exposed to enhanced electrochemiluminescence (Thermo Fisher Science) to develop protein bands. Data analysis was performed using Image J software (NIH, Bethesda) [36].

### RNA immunoprecipitation (RIP) test

BMSCs were transfected with pMS2bp-GFP and MS2, MS2-HAGLR, or MS2-mutant (MUT)-HAGLR and collected after 48 h. Biotin-conjugated RNA complexes were pulled down and checked for RIP according to the manufacturer's instructions using the Magna RIP kit (Millipore, USA). Samples were incubated with anti-GFP and anti-immunoglobulin G, and miR-182-5p expression was detected by RT-qPCR [37].

### RNA pull-down test

BMSCs were transfected with biotinylated miR-182-5p and HAGLR. After 72 h, BMSCs were collected, and cell lysates were bound to M-280 streptavidin magnetic beads (Sigma) to pull down the biotin-conjugated RNA complexes. Then, the RNA-bound beads were purified with TRIzol. HAGLR or miR-182-5p expression was tested by RT-qPCR.

### Determination of luciferase activity

Wild-type (WT) or MUT HAGLR binding to miR-182-5p was subcloned into the pGL3 vector. BMSCs were co-transfected with miR-182-5p (RiboBio, Guangzhou, China) and 10 μg of pur-WT-HAGLR or pur-MUT-HAGLR. WT or MUT Hoxa10 and miR-182-5p were subcloned into a pGL3-based vector (Promega). miR-182-5p (RiboBio) was co-transfected with 10 μg pluco-WT-Hoxa10 or pluco-MUT-Hoxa10. After transfection of 48 h, a test of luciferase activity was done via the dual luciferase detection system (Promega Corporation, Fitchburg, WI, USA) [38].

### Statistical analysis

Data analysis was performed using SPSS 21.0 (SPSS, Inc, Chicago, IL, USA) statistical software. After the Kolmogorov–Smirnov test, the data were normally distributed, and the results were expressed as mean ± standard deviation (SD). Two-group comparisons were done with t test and comparisons among multiple groups were done with one-way analysis of variance (ANOVA) and Fisher’s least significant difference t-test. The enumeration data were expressed as rates or percentages, and the chi-square test was used for comparative analysis. *P* was a two-sided test, and *P* < 0.05 was accepted as indicative of distinct differences.

## Results

### HAGLR, miR-182-5p and Hoxa10 in PMOP

**Fig. 1:**
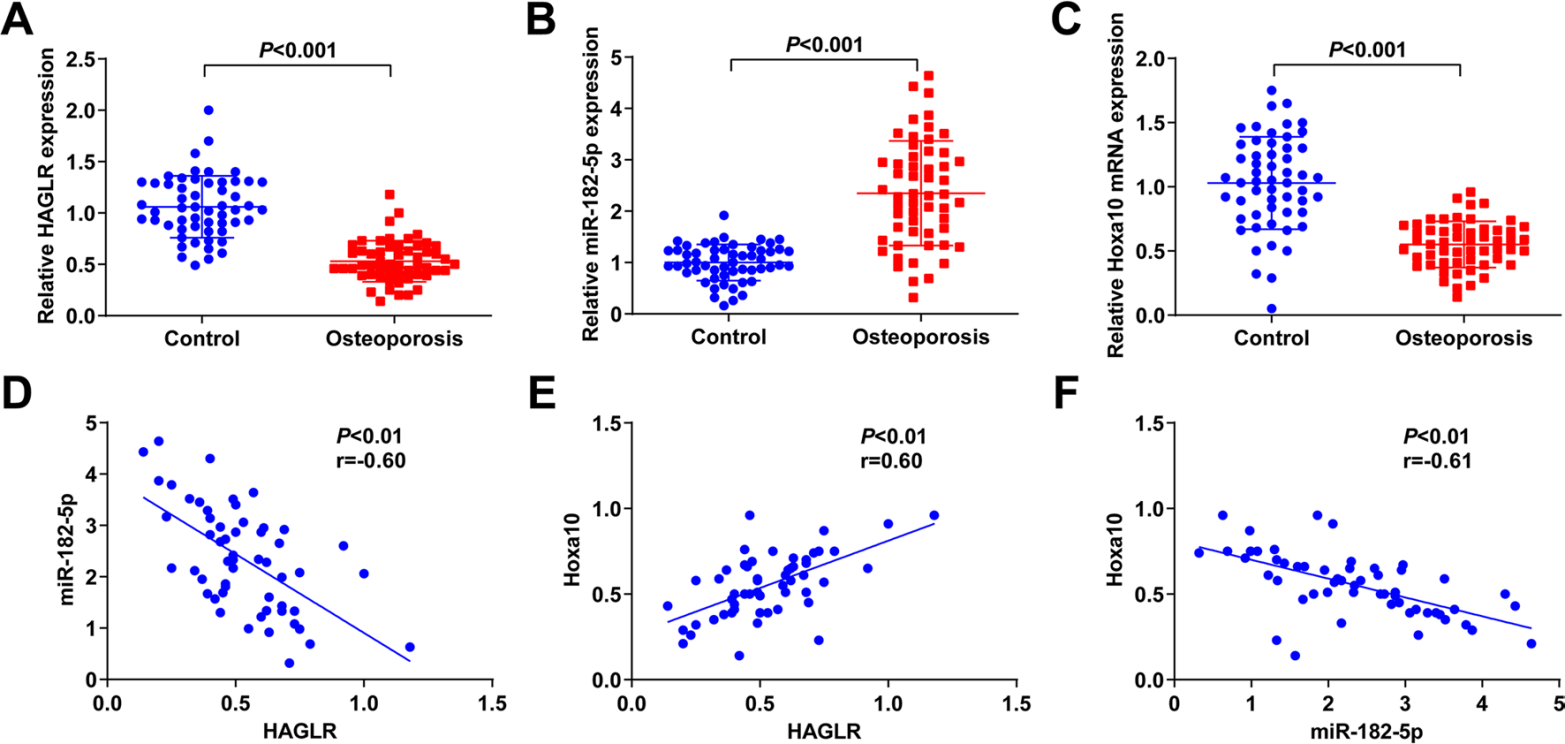
HAGLR, miR-182-5p and Hoxa10 in PMOP patients. **A–C** RT-qPCR detection of HAGLR, miR-182-5p, and Hoxa10 in the peripheral blood of PMOP patients (*n* = 55) and healthy PM women (control, *n* = 55); **D** PMOP patients. Pearson linear regression analysis of association of HAGLR with miR-182-5p (*n* = 55); **E** PMOP patients. The association of HAGLR with Hoxa10 (*n* = 55); (**F**) PMOP patients. Relevance of miR-182-5p with Hoxa10 (*n* = 55)

To explore the biological functions of HAGLR, miR-182-5p, and Hoxa10 in PMOP, their expression levels in the peripheral blood of PMOP patients and healthy PM women were tested by RT-qPCR. HAGLR and Hoxa10 were down-regulated and miR-182-5p was elevated in PMOP patients compared with healthy controls (Fig. 1A–C). Pearson linear regression analysis noted that HAGLR was negatively linked with miR-182-5p and positively associated with Hoxa10, while miR-182-5p was negatively associated with Hoxa10 (Fig. 1D–F).

### The association of HAGLR/miR-182-5p with the osteogenic induction time of BMSCs

**Fig. 2:**
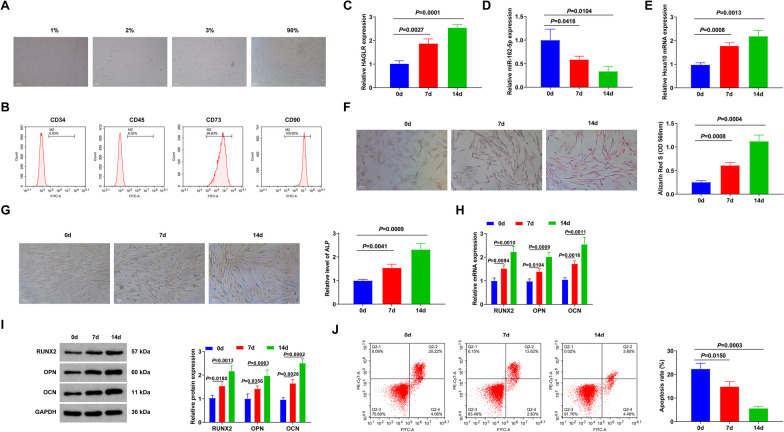
The association of HAGLR/miR-182-5p with osteogenic induction time of BMSCs. **A** Separation of BMSCs from mouse bone marrow tissue, and observation of BMSC morphology under the microscope; **B** Flow cytometry examination of the positive rate of CD45/90/34/73 in BMSCs; **C**–**E** RT-qPCR examination of HAGLR and miR-182-5p in BMSCs after stimulation of 23 d; **F** Alizarin red S staining test of cell calcium deposition; **G** ALP staining results of osteoblasts; **H**–**I** RT-qPCR or Western Blot examination of OD-associated genes RUNX2, OPN and OCN; **J** Flow cytometry test of cell apoptosis. *N* = 3

BMSCs were isolated from mouse bone marrow. After 1 week of primary culture, BMSCs were polygonal with a limited distribution area. The second-generation cells were long spindle fibroblasts with large nuclei and abundant cytoplasm. The third generation is typical of bipolar spindle cells. When the confluence reached 90%, the cells were observed to be spirally shaped (Fig. 2A). Additionally, flow cytometry identified that BMSCs (the third generation) were positive for CD73 and CD90 and negative for CD34 and CD45 (Fig. 2B). These results confirmed that the cells isolated from mice were BMSCs.

Subsequently, BMSCs were induced, RT-qPCR tested HAGLR, miR-182-5p, and Hoxa10 at 0, 7, and 14 d. HAGLR and Hoxa10 levels were augmented, while miR-182-5p was suppressed time-dependently (Fig. 2C–E). Alizarin red S staining showed manifested intense staining of BMSCs and BMSC mineralization (Fig. 2F). ALP staining showed increased ALP expression (Fig. 2G). Additionally, mRNA and protein expressions of OD-associated genes RUNX2, OPN, and OCN were gradually elevated as well (Fig. 2H–I). Flow cytometry revealed that with the increase of OD degree of BMSCs, apoptosis decreased significantly on days 7 and 14 (Fig. 2J). These experiments suggest that HAGLR/miR-182-5p may be involved in BMSC OD.

### Silenced HAGLR restrains BMSC OD

**Fig. 3:**
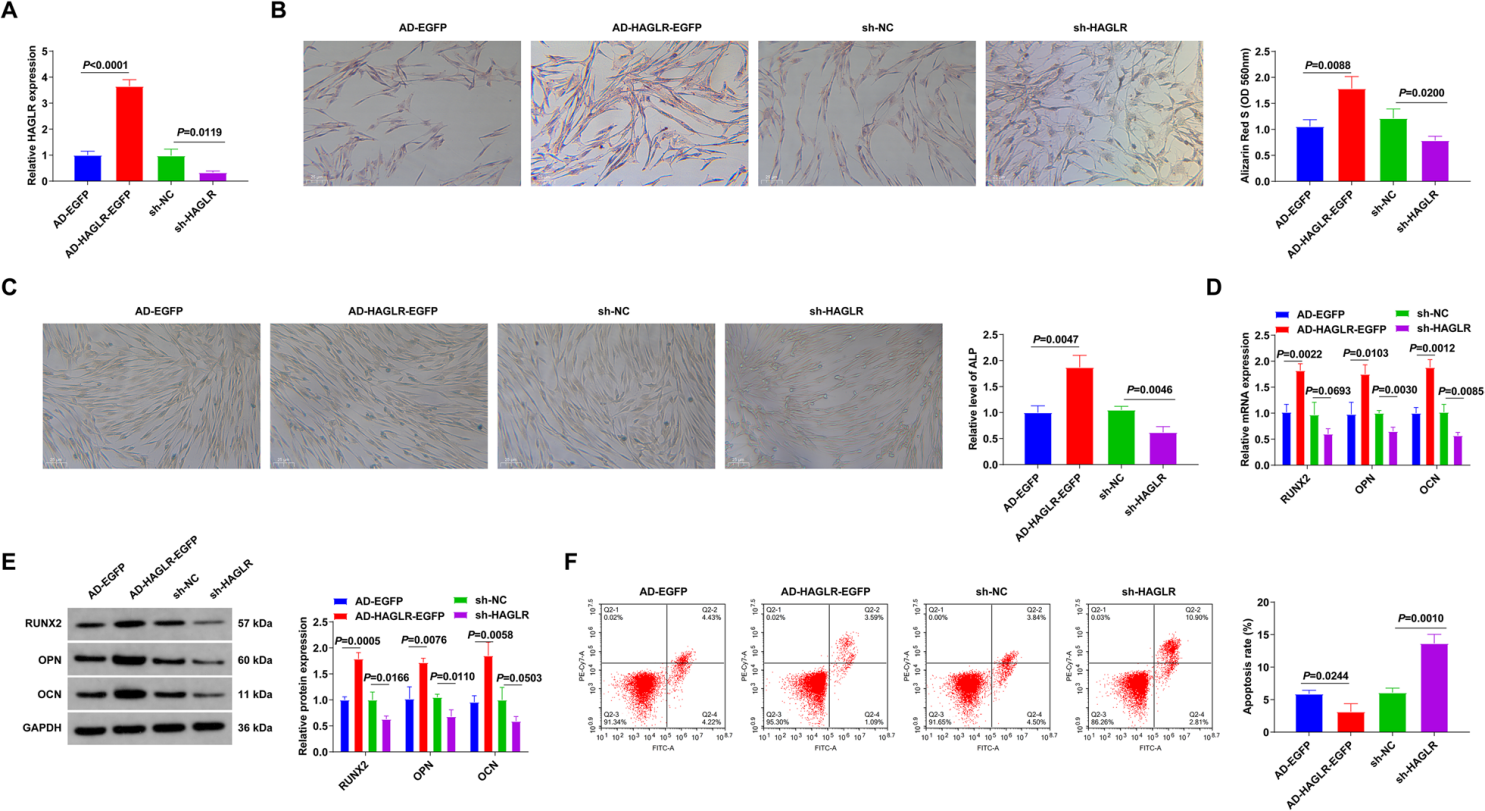
Repressive HAGLR constrains BMSC OD. **A** RT-qPCR detection of HAGLR; **B** Alizarin Red S staining test of BMSC mineralization ability; **C** ALP activity in BMSCs; **D–E** RT-qPCR or Western Blot examination of osteogenic markers like OPN, OCN and Runx2 in BMSCs; **F** Flow cytometry test of BMSC apoptosis. **A**–**F** After silencing or elevating HAGLR. *N* = 3

On the one hand, to determine the role of HAGLR in OD, recombinant vectors were transfected to increase HAGLR. On the other hand, HAGLR gene-silencing BMSCs were induced. The transfection efficiency was verified by RT-qPCR (Fig. 3A). Silenced HAGLR constrained BMSC mineralization ability, while elevating HAGLR had the opposite effects (Fig. 3B). In the meantime, during BMSC OD, ALP content was decreased after silencing HAGLR, while it was augmented after elevating HAGLR (Fig. 3C). Additionally, silencing HAGLR decreased expressions of osteogenic markers (OPN, OCN, and RUNX2), whereas overexpressing HAGLR elevated expressions of these markers (Fig. 3D, E). Repression of HAGLR strengthened BMSC apoptosis, while elevated HAGLR suppressed cell apoptosis (Fig. 3F). These results suggest that HAGLR silencing inhibits OD of BMSCs.

### HAGLR performs as a sponge for miR-182-5p

**Fig. 4:**
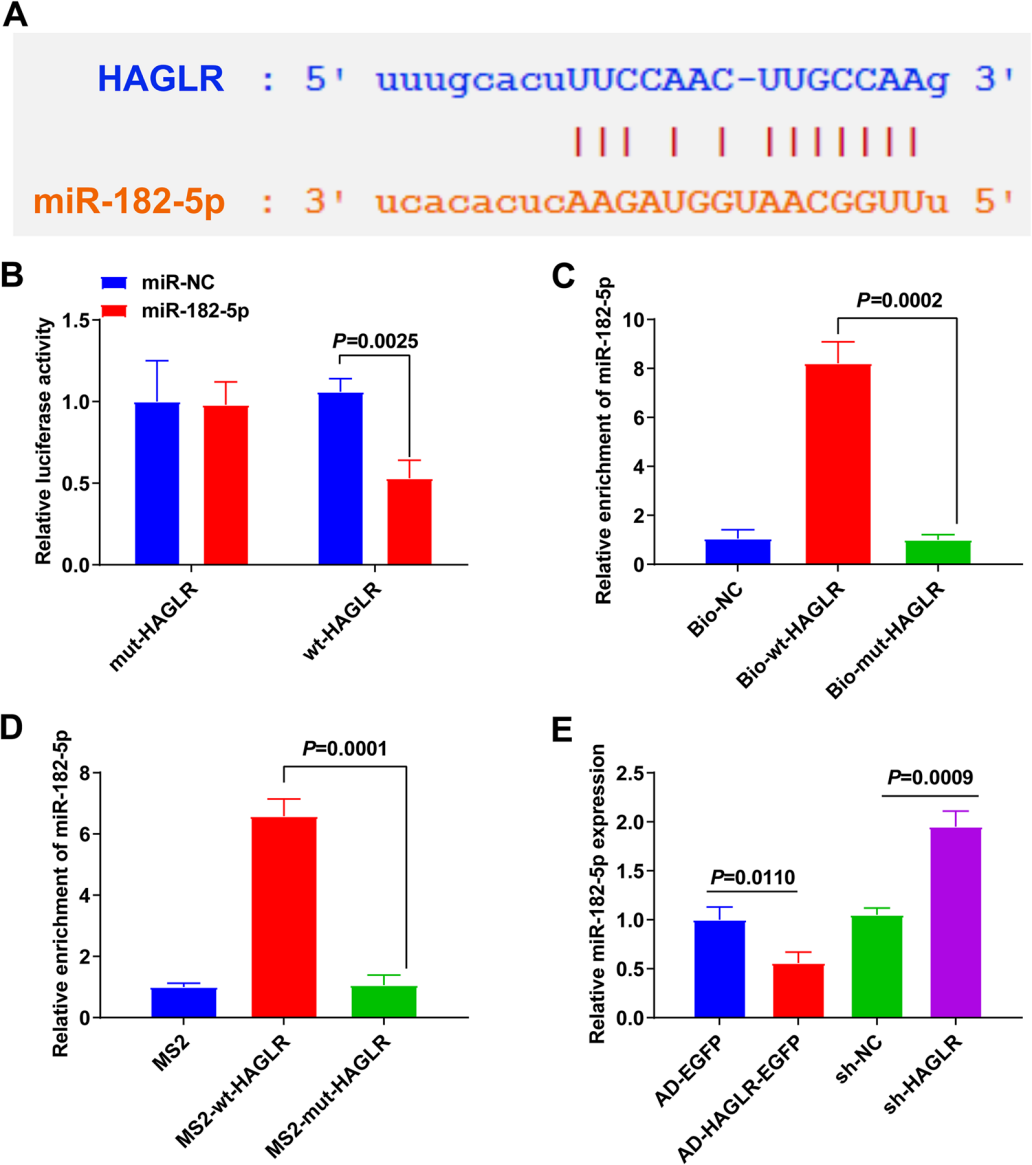
HAGLR performs as a sponge for miR-182-5p. **A** The bioinformatics website forecast of the binding site of miR-182-5p with HAGLR; **B** The luciferase activity assay test of the targeting of miR-182-5p with HAGLR; **C**, **D** RNA Pull down and RIP experiments; **E** Detection of miR-182-5p in BMSC with repressive or elevated HAGLR. *N* = 3

Possible miRNA binding to HAGLR was predicted by StarBase. As shown in Fig. 4A, HAGLR may target miR-182-5p. Luciferase assay was used to determine the target interaction between HAGLR and miR-182-5p. As shown in Fig. 4B, miR-182-5p decreased the luciferase activity of WT-HAGLR but had no effect on the luciferase activity of MUT-HAGLR. RNA pull-down assay and RIP assay further verified the relationship between miR-182-5p and HAGLR. As manifested in Fig. 4C, miR-182-5p was decreased by biotin-labeled WT-HAGLR but not MUT-HAGLR. MiR-182-5p was abundant in the HAGLR group but not in the mut-HAGLR group (Fig. 4D). Besides, suppression of HAGLR augmented miR-182-5p expression, while elevation of HAGLR reduced miR-182-5p expression (Fig. 4E). In short, HAGLR performed as a sponge for miR-182-5p to suppress miR-182-5p expression.

### Suppression of miR-182-5p ameliorates BMSC OD

**Fig. 5:**
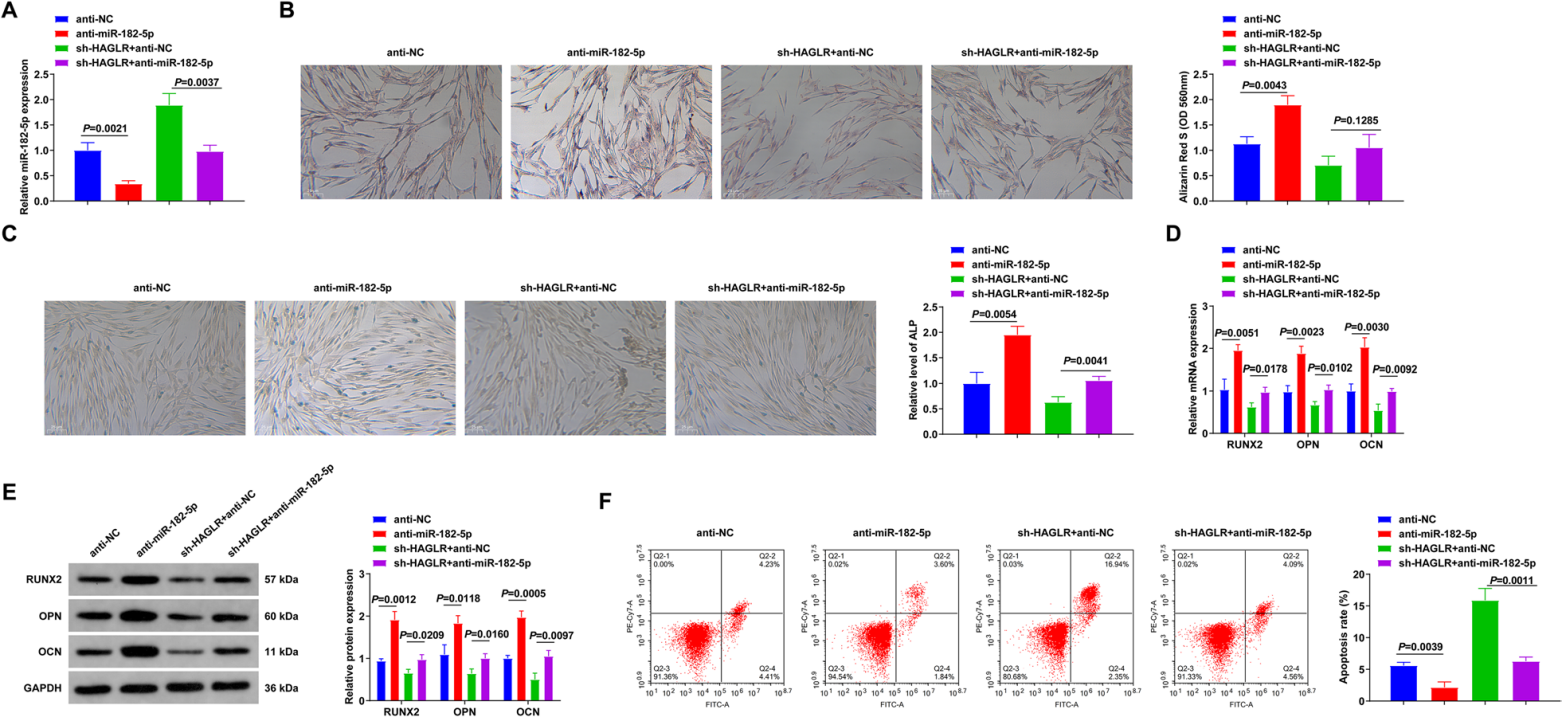
Repressive miR-182-5p accelerates BMSC OD. **A** RT-qPCR examination of HAGLR; **B** Alizarin Red S staining test of BMSC mineralization ability; **C** ALP activity in BMSCs; **D**, **E** RT-qPCR or Western Blot detection of osteogenic markers like OPN, OCN and Runx2 in BMSCs; **F** Flow cytometry test of BMSC apoptosis; *N* = 3

BMSCs were transfected with anti-NC or anti-miR-182-5p to explore the role of miR-182-5p in BMSC OD. In addition, miR-182-5p mimic was transfected into HAGLR-silenced BMSCs to further clarify the association between miR-182-5p and HAGLR (Fig. 5A). The experiment manifested that suppression of miR-182-5p boosted BMSC mineralization ability, elevated ALP content, and increased mRNA and protein expressions of OPN, OCN, and RUNX2 (Fig. 5B–E). Suppression of miR-182-5p decreased BMSC apoptosis (Fig. 5F). Additionally, repression of miR-182-5p turned around the effects of silenced HAGLR on BMSC OD (Fig. 5B–F). To sum up, suppression of miR-182-5p ameliorated BMSC OD.

### MiR-182-5p targets Hoxa10

**Fig. 6:**
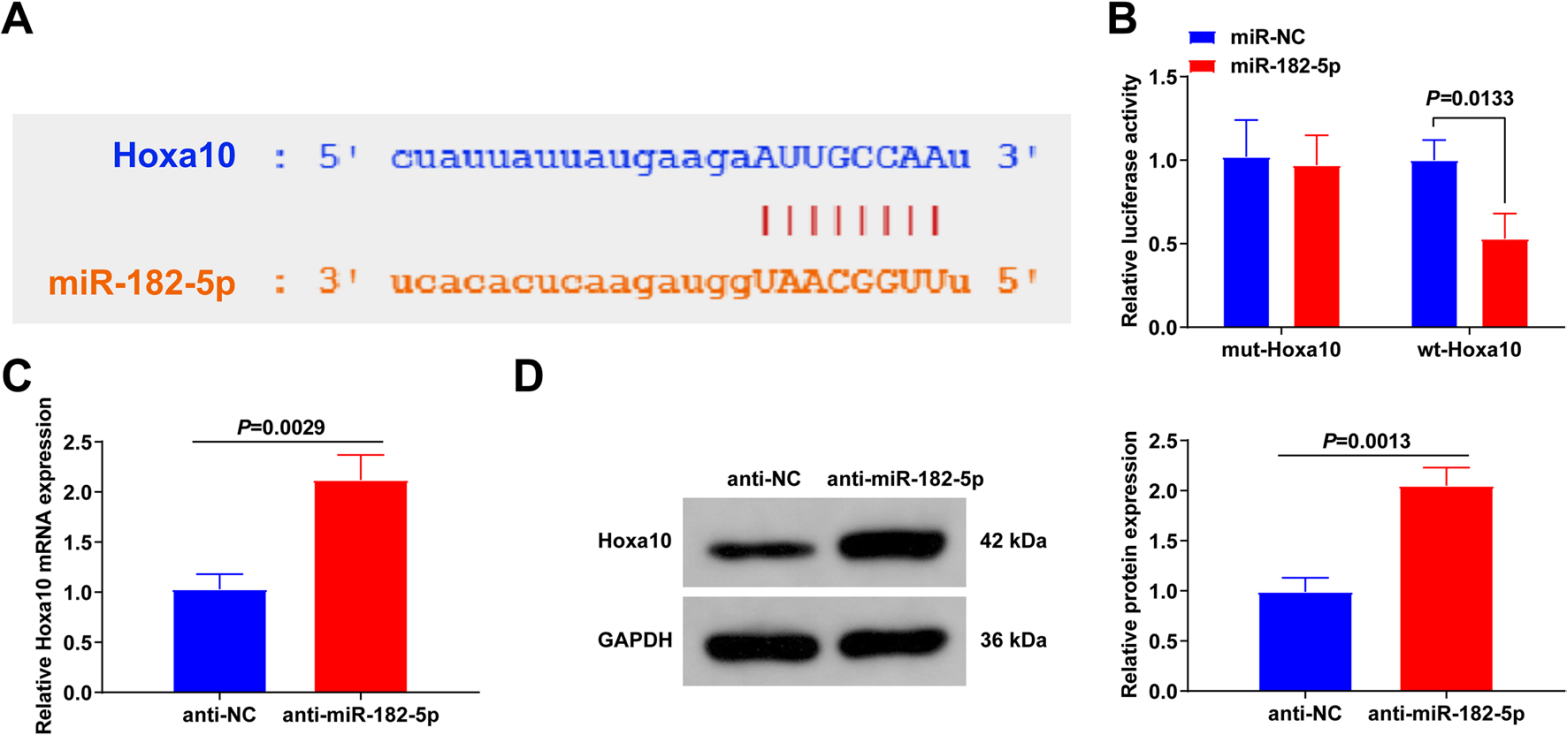
MiR-182-5p targets Hoxa10. **A** The bioinformatics website reveal of the predicted binding sites of Hoxa10 with miR-182-5p; **B** The luciferase activity assay illumination of co-transfection of miR-182-5p mimic and Hoxa10-Wt being available to elevate the luciferase activity of BMSCs vs. other groups; **C** RT-qPCR test of Hoxa10 mRNA in BMSCs with suppressive miR-182-5p; **D** Western Blot examination of Hoxa10 in BMSCs with repressive miR-182-5p. *N* = 3

Bioinformatics analysis clarified that miR-182-5p and Hoxa10 had complementary binding sites (Fig. 6A). Co-transfection of miR-182-5p and wt-Hoxa10 repressed the luciferase activity (Fig. 6B). Elevated Hoxa10 mRNA (Fig. 6C) and protein (Fig. 6D) expression was detected in BMSCs knocking down miR-182-5p. These results indicate that miR-182-5p negatively regulates Hoxa10 expression.

### Elevated HAGLR alleviates PMOP

**Fig. 7:**
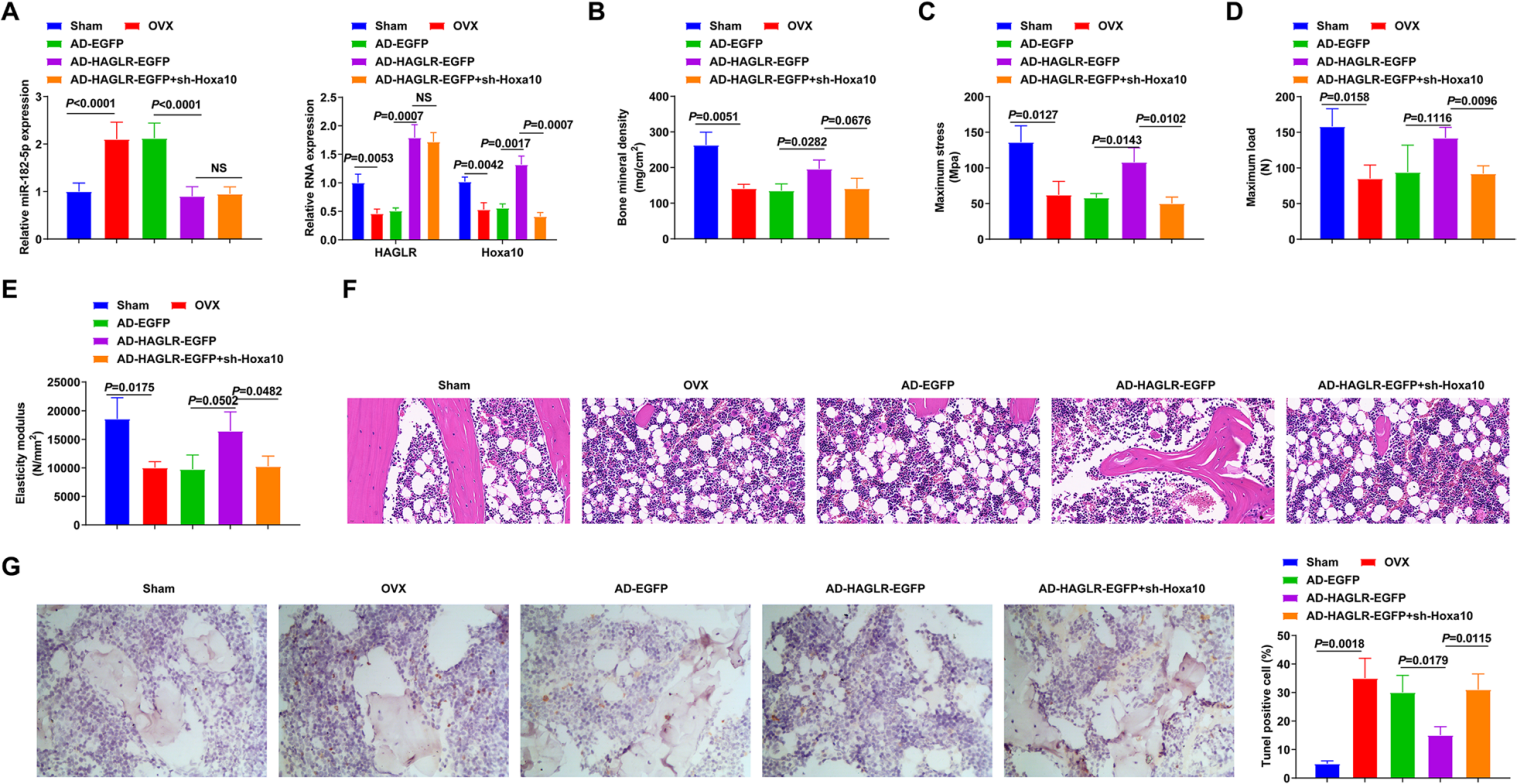
Elevated HAGLR or repressive miR-182-5p alleviates PMOP. **A** RT-qPCR detection of HAGLR, miR-182-5p, and Hoxa10 in the OVX mice; **B** Femur BMD; **C** Femur’s maximum bending stress; **D** Femur’s maximum load; **E** Femur’s Elastic modulus; **F** H&E staining reveal of histopathological conditions; **G** TUNEL staining examination of chondrocyte apoptosis in articular cartilage. *n* = 8

To further verify that HAGLR alleviates PMOP, OVX mice were constructed and injected with the corresponding lentivirus (Fig. 7A). Compared with Sham mice, OVX mice showed a decrease in BMD, elastic modulus, maximum load, and maximum bending stress, confirming that the model was successfully constructed. These indices increased after overexpression of HAGLR. Furthermore, BMD and three biomechanical parameters decreased after sh-Hoxa10 injection (Fig. 7B–E), These results indicated that Hoxa10 is involved in HAGLR/miR-182-5p axis to participate in OVX mouse development. HE staining showed that the subchondral trabecular bone volume decreased in OVX mice. After overexpressing HAGLR, the bone cortex was thickened, the bone trabecular was increased, and the intertrabecular connectivity was better (Fig. 7F). TUNEL assay manifested that elevated HAGLR reduced TUNEL-positive chondrocytes in OVX mice, while sh-Hoxa10 turned around the effect of elevated HAGLR on chondrocyte apoptosis (Fig. 7G). In summary, overexpression of HAGLR alleviates PMOP, and down-regulation of Hoxa10 reverses the effect of overexpression of HAGLR.

## Discussion

BMSCs are multifunctional cells, being available to differentiate into osteogenic, adipogenic, and chondrogenic directions [39]. BMSC activity is nearly associated with PMOP occurrence and progression [40]. Consequently, it was crucial to understand the mechanism of BMSC OD for the development of novel PMOP treatment strategies. LncRNA, a critical gene regulator, is linked with multiple bone diseases. In this research, HAGLR was decreased in PMOP patients’ peripheral blood, illuminating that HAGLR might be implicated in PMOP. Additionally, HAGLR was gradually elevated during BMSC OD. Repression of HAGLR restrained the OD of BMSCs in *vitro*, while augmented HAGLR oppositely acted. In the meantime, elevated HAGLR suppressed PMOP progression in *vivo*. These results elaborated that augmented HAGLR suppressed PMOP via ameliorating BMSC proliferation and OD.

BMSCs exerted a crucial action in PMOP progression. Differentiation of BMSCs into osteoblasts was critical for maintaining normal BMD and modulating bone formation [41]. Numerous studies have elucidated that lncRNA participates in mediating BMSC differentiation. For instance, lncRNA HOTAIR is augmented in the serum of OP patients and suppresses BMSC OD via modulating the Wnt/β-catenin pathway [42]. LncRNA H19 is decreased in PMOP, while augmented H19 restrains BMSC proliferation and OD via silencing miR-19b-3p [43]. HAGLR is HOXD antisense growth-associated LncRNA, which has been testified to be associated with diversified diseases, covering cancer [44], neurodegenerative diseases [45], heart disease [46], and bone disease [47]. Suppression of HAGLR constrains the healing of femoral neck fractures via suppressing osteoblast growth. In this research, HAGLR was decreased in PMOP tissues, and elevated HAGLR boosted BMSC proliferation and OD in *vivo* and in *vitro*. These findings manifested that HAGLR might perform as the latent target for PMOP therapy.

Typically, LncRNA prevalently performs as a sponge of miRNA to modulate protein translation and cell activity [48]. In tibial fractures, HAGLR exerts a protective role by serving as a sponge of miR-214-3p to boost BMP2. In this study, miR-182-5p was elevated in PMOP and negatively linked with HAGLR. HAGLR had a targeting relationship with miR-182-5p in PMOP. miR-182-5p has been discovered to be elevated in OP patients’ femur tissue, while decreased miR-182-5p activates the Rap1/MAPK pathway via targeting ADCY6, thereby boosting the differentiation of osteoblasts [49]. Additionally, elevated miR-182-5p restrains chondrogenic differentiation of BMSCs via silencing parathyroid hormone-like hormone [50]. Nevertheless, decreased miR-182-5p turned around the effects of silenced HAGLR on BMSCs, and boosted the OD of BMSCs. Additionally, miR-182-5p negatively modulated Hoxa10 to restrain BMSC OD.

Hoxa10 is a transcription factor covering a homeobox and belongs to the HOX family, which is a crucial regulator of the osteogenic process and is available to control osteoblast production via immediately activating bone regulatory and phenotypic genes [51]. Recently, studies have shown that the OD of Hoxa10 and BMSCs is elevated in osteogenic induction and is regulated by miRNAs. Enhanced HOXA10 improves the OD of BMSCs [52]. In this research, Hoxa10 was gradually elevated during BMSC OD, while repression of Hoxa10 turned around the action of augmented HAGLR or silenced miR-182-5p on BMSC OD.

Admittedly, this study has some limitations. After testing, HAGLR in the peripheral blood of PMOP patients is reduced, but the association of HAGLR and PMOP should be verified by a large number of people, which provides a reliable molecular biomarker for PMOP diagnosis.

## Conclusion

In short, this study is the first to elucidate the effect of HAGLR on PMOP by regulating the OD of BMSCs. The mechanism of the HAGLR/miR-182-5p/Hoxa10 axis in PMOP was preliminarily explained, and the molecular mechanism by which HAGLR mediated the regulation of OD of BMSCs by Hoxa10 by acting as a sponge for miR-182-5p was revealed to alleviate PMOP. This study opens up a new avenue for PMOP prevention and provides a rationale for developing new therapies targeting HAGLR.

## Data Availability

The data are available from the corresponding author upon request.
